# Parents’ Awareness of Infectious Diseases Epidemiology in Poland and Ukraine

**DOI:** 10.3390/healthcare12121199

**Published:** 2024-06-14

**Authors:** Maciej Stępień, Martyna Cholewik, Jan Żuromski, Carlo Bieńkowski, Maria Pokorska-Śpiewak

**Affiliations:** 1 Student Scientific Circle at the Department of Children’s Infectious Diseases, Medical University of Warsaw, Wolska 37, 01-201 Warsaw, Poland; s073965@student.wum.edu.pl (M.S.); martyna.cholewik@student.wum.edu.pl (M.C.); 2 Leiden Institute of Advanced Computer Science, Leiden University of Leiden, Einsteinweg 55, 2333 CC Leiden, The Netherlands; j.p.zuromski@umail.leidenuniv.nl; 3 Department of Children’s Infectious Diseases, Medical University of Warsaw, Wolska 37, 02-091 Warsaw, Poland; maria.pokorska-spiewak@wum.edu.pl; 4 Hospital of Infectious Diseases, 01-201 Warsaw, Poland

**Keywords:** infectious diseases, Ukraine, international migration, vaccination coverage

## Abstract

Background: There has been a decline in vaccine-related confidence in Ukraine over the past few years, resulting in high rates of infectious diseases. Due to the arrival of a large number of refugees in Poland following the outbreak of war in Ukraine in February 2022, the risk of infectious diseases in Poland among children and adults has increased. The present study aimed to analyze the relationship between parents’ knowledge of the epidemiological situation of infectious diseases in Poland and Ukraine and socio-demographic factors and their attitudes towards vaccination. Material and methods: A cross-sectional survey study was designed. Data were collected through an online questionnaire between November 2022 and January 2023, where the inclusion criteria was having a child under 18 years of age. Study participants’ stratification was based on responses to seven knowledge questions; those who answered all questions correctly were allocated to group 1, and the others to group 2 for further analysis. Results: A total of 547 parents were included. Their median age was 34 years [IQR: 31–39]. There were 233 (42.60%) participants who answered all the knowledge questions correctly. Respondents from group 1 were older (*p* = 0.033), were more likely to be in favor of recommended vaccination (*p* = 0.040), to be vaccinated with four doses against SARS-CoV-2 (*p* = 0.014), to have their children vaccinated against SARS-CoV-2 (*p* < 0.001), and to believe that the influx of migrants from Ukraine would increase the incidence rate of infectious diseases in Poland in the future (*p* < 0.001). They also declared awareness of the impact of migration on the epidemiological situation in Poland (*p* < 0.001) more often. According to the logistic regression model, older parents (OR = 1.31, *p* = 0.029), those who were willing to receive additional vaccinations due to migration (OR = 4.29, *p* = 0.003), those who were aware of the impact of migration on the epidemiological situation (OR = 2.38, *p* < 0.001), and those who believed that migration would have affected the incidence rate of infectious diseases in Poland (OR = 2.28, *p* = 0.003) were significantly more likely to belong to group 1. However, parents who were willing to vaccinate their children with additional vaccinations due to migration were significantly less likely to answer all the questions correctly (OR = 0.21, *p* = 0.002). Conclusions: Awareness of the epidemiological situation in Poland and Ukraine among parents is related to a greater awareness of the impact of migration and the use of recommended vaccinations and those against SARS-CoV-2. Additional vaccinations should be further promoted among both Poles and migrants.

## 1. Introduction

Ukraine is a country where vaccination rates against many infectious diseases, such as poliomyelitis, measles, tuberculosis, and COVID-19, are significantly lower than in other European Union countries [1,2,3]. Before the start of the war in Ukraine in February 2022, approximately 1.35 million Ukrainians were living in Poland. After the outbreak of the war, this number surged. Between February 2022 and May 2022, over 3.5 million refugees from Ukraine crossed the Polish border, predominantly women and children [4]. While parents from both Ukraine and Poland show higher levels of vaccine hesitancy compared to other European countries [5,6], conflicts in Ukraine in recent years have led to a series of disinformation activities in the region, which led to an additional decline in trust in health services and vaccination [7]. According to the European Centre for Disease Prevention and Control, the incidence of some infectious diseases in Ukraine over the past few years has been higher than in Poland [5,6]. This hesitancy and subsequent rise in incidence have an outsized influence on children. The most notable differences relate to measles, polio, tuberculosis, and COVID-19 [5]. In 2010, vaccination coverage with two doses of measles, mumps, and rubella (MMR) vaccine in Ukraine reached only 41%, resulting in a sharp increase in the number of measles cases in subsequent years. In 2017, the number of cases increased 47-fold compared to the preceding year, and in 2019, the number exceeded 57,000 [2]. In Poland, measles cases are also reported annually, mostly in unvaccinated individuals, and a high proportion of them develop bacterial complications, including pneumonia and otitis [8]. In Ukraine, vaccination rates with the three-dose polio vaccine decreased from 91% in 2008 to 15% in mid-2015. This decline may have been the reason for two unrelated cases of polio reported in 2015, when paralysis was observed in unvaccinated children due to the circulating vaccine-derived poliovirus type 1. Despite Europe being declared a polio-free area in 2002, an identified poliomyelitis case of an unvaccinated 17 month old girl emerged in 2021 in Ukraine [2]. Before the outbreak of war, Ukraine had the fourth-highest incidence of tuberculosis in the European region and the fifth-highest number of drug-resistant tuberculosis cases in the world [9]. The Ukrainian vaccination rate against SARS-CoV-2 on the day the war broke out equated to only 34% [2]. Ukrainian refugees often refuse to receive additional vaccinations, despite the offer of free care in the new country [10]. Despite the fact that the general population of Poland is well protected against infectious diseases through adequate vaccination levels and herd immunity, geographical clusters with high proportions of unvaccinated migrants may lead to outbreaks [11,12]. This new epidemiologic challenge requires acute attention to avoid further outbrakes of diseases, especially those that are preventable through vaccination. Regarding Ukrainian migrants, the poor vaccination coverage is probably a combination of various factors, such as the general difficulty in obtaining information on vaccination and the lack of trust in Ukrainian government institutions and health professionals, which in turn influences the negative opinion on the quality, safety, and relevance of vaccines [13,14]. One of the major steps of combating this crisis seems therefore to be educating parents. However, the relationship between the level of knowledge on vaccination and acceptance of vaccines is not clear-cut. Overall, there is good, strong evidence pointing to a correlation between increased knowledge of vaccination and uptake. However, due to conformism, some people with limited knowledge are less likely to oppose the legitimacy of vaccines [15,16]. Therefore, there is a need to conduct more research on the subject. This study aimed to analyze the relationship between knowledge of the epidemiological situation in Poland and Ukraine versus socio-demographic factors and parents’ attitudes toward vaccination.

## 2. Material and Methods

### 2.1. Study Design and Participants

A cross-sectional survey was designed for parents, collecting socio-demographic data, their attitudes towards vaccination, their knowledge of the current epidemiological situation in Poland, and their willingness to carry out additional vaccinations. The survey consisted of 25 single-choice and 4 multiple-choice questions. The classification of vaccinations into mandatory and recommended was based on the law regulating the immunization program in Poland in 2023 [17].

“Google Forms” was used to collect data via an online questionnaire between 15 November 2022 and 4 January 2023. The survey was shared on social media (Facebook, Instagram) and on forums and groups related to parenting issues. Each participant was asked to complete the survey only once. There was no time limit to access the form. At the end of completing the survey, participants were provided with information on the epidemiological situation in Ukraine and Poland for educational purposes.

Based on the responses, the study group was divided into two subgroups for further analysis. Stratification was based on responses to seven knowledge questions. The questions related to the comparison of vaccination calendar, vaccination and incidence rates in Poland and Ukraine, and separately covered mandatory, recommended, and SARS-CoV-2 vaccination. Those who answered all 7 questions correctly were classified as group 1. Correct answers were defined as those that acknowledged that Poles get vaccinated more frequently with mandatory, recommended, and SARS-CoV-2 vaccinations, and that Ukrainians suffer more frequently from COVID-19 and diseases covered by recommended and mandatory vaccinations. All others (with a minimum of one incorrect answer) were classified as group 2. We have chosen to make a division into these two groups because we wanted to explore how the level of knowledge depends on different socio-demographic factors and attitudes towards the epidemiological situation among parents. Our aim was to determine not only how those with the highest levels of knowledge respond, but whether there are factors that characterize these individuals and differentiate them from the population that does not have such knowledge. In our view, the search for such factors is important in order to know what to base efforts to increase vaccination rates among the population on.

### 2.2. Inclusion Criteria

Inclusion criteria included completing the form and being the parent of a child under the age of 18. Each participant agreed to participate anonymously in the study and consented to the publication of the results of this study by completing the form.

### 2.3. Exclusion Criteria

Twenty-one questionnaires have been rejected due to respondents indicating that they were not parents of at least one child under the age of 18.

### 2.4. Statistical Analysis and Descriptive Statistics Methods

Group 1 and group 2 were compared in two ways. In the first step, a Pearson chi-square test was performed to compare categorical variables, and a Mann–Whitney U-test was used to assess continuous variables. A *p*-value of < 0.05 was considered significant. Python’s SciPy library (version 1.11.1) and Quick Statistics Calculators were used to perform statistical analysis [18].

In the second stage, a binary logistic regression was performed. Membership of group 1, meaning a correct answer to the 7 knowledge questions, was defined as the dependent variable. In order to suit the requirements of the model, the dataset was modified to only include numerical values. This was conducted by either converting the answers to a numerical scale (e.g., No impact = 0, Wanted to get vaccinated = 1, Already vaccinated = 2) or by introducing one-hot encoding (e.g., gender variable with answer options male/female/other was converted to three yes/no variables). The resulting dataset was divided into train and test sets, with an 80:20 ratio to prevent overfitting. The logistic regression was performed using the statsmodels Python library (version 0.14.0). The subsequent analysis involved evaluating the model using the Area Under the Receiver Operating Characteristic Curve (AUC) metric, accuracy, recall, and the confusion matrix. All evaluations were performed on the test set. Additionally, the influence of individual variables on the predicted outcome was analyzed.

### 2.5. Ethical Statement

The study was approved by the Medical University of Warsaw’s Bioethics Committee. Approval number: AKBE/6/2023. The study was conducted in accordance with the Declaration of Helsinki.

## 3. Results

**Figure 1:**
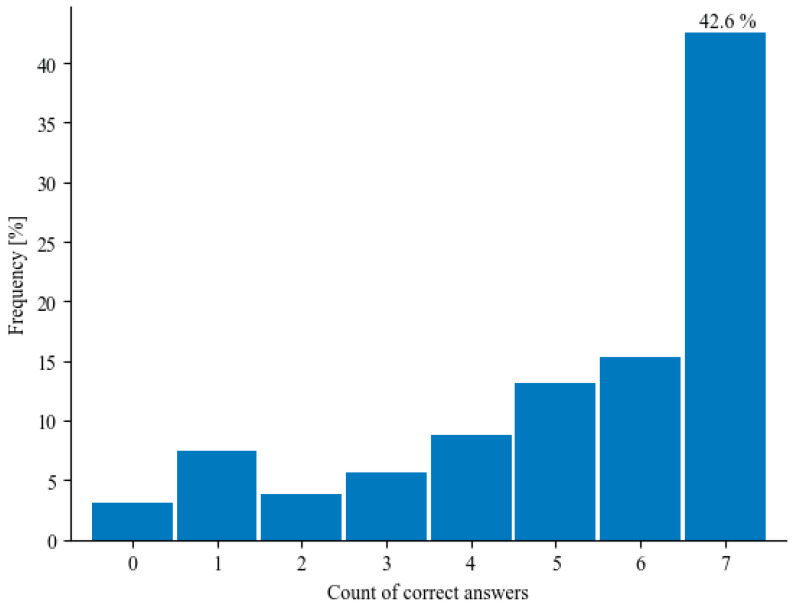
Distribution of the count of correct answers for the seven questions from the knowledge section of the questionnaire. The distribution indicates a visible division into two groups: respondents who answered all seven questions correctly vs. those who did not.

A total of 547 questionnaires were collected and met the inclusion criteria. The study participants’ median age was 34 [interquartile range (IQR): 31–39 years]. There were 233 (42.60%) participants who answered all of the knowledge questions correctly and were assigned to Group 1 (see Figure 1).

Respondents with greater knowledge of the epidemiological situation in Poland and Ukraine were significantly older (35 years [IQR: 31–39] vs. 34 years [IQR: 30–38], *p* = 0.033), were more likely to be in favor on usage of recommended vaccination (213/233, 93.13% vs. 269/314, 85.67%, *p* = 0.040), were more inclined to be vaccinated with four doses against SARS-CoV-2 (52/233, 22.32% vs. 47/314, 14.97%, *p* = 0.014), more often vaccinated their children against SARS-CoV-2 (194/233, 83.26% vs. 222/314, 70.70%, *p* = 0 < 0.001), and were more likely to believe that the influx of migrants from Ukraine would increase the incidence rate of infectious diseases in Poland in the future (200/233, 85.84% vs. 196/314, 62.42%, *p* < 0.001). They also more often declared an awareness of the impact of the influx of immigrants on the epidemiological situation in Poland (164/233, 70.39% vs. 147/314, 46.82%, *p* < 0.001). These results are shown in Table 1.

**Figure 2:**
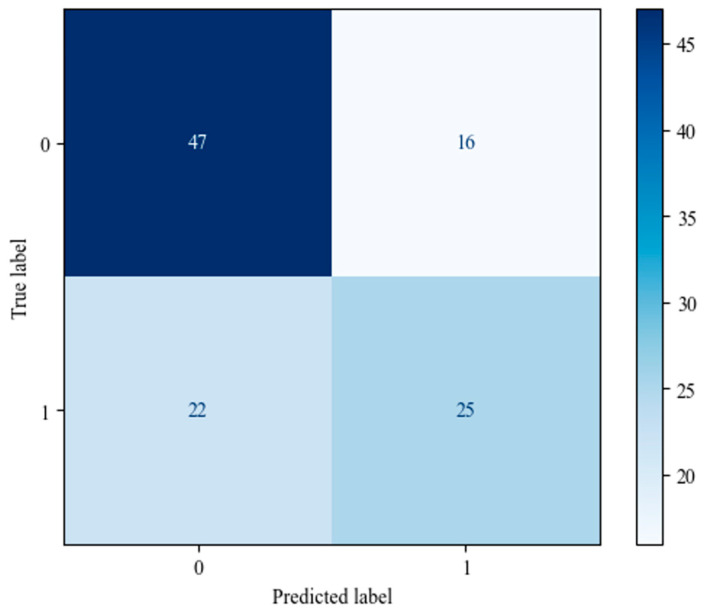
Confusion matrix for the logistic regression model showing how the regression model (Predicted labels) classified the test samples in relation to their actual values (True label). Individuals who answered all of the questions correctly correspond to ‘1’s in the graph; all other individuals correspond to ‘0’s.

Training a logistic regression model on the dataset resulted in an AUC of 71.6%. Analysis of the confusion matrix (see Figure 2) shows that the model struggled to capture positive observations; out of the 47 people in the test set that answered all of the questions correctly, only 25 were classified that way by the model (recall of 53.2%). Conversely, the model was mostly able to identify observations that did not answer all of the questions correctly. In this case, out of the 63 ‘negative’ observations in the test set, the model correctly classified 47 of them (accuracy of 65.5%). The model indicated five statistically significant variables:

- Impact of the study on the administration of additional vaccinations for their children.
- Impact of the study on the administration of additional vaccinations.
- Opinion on the impact of the influx of migrants from Ukraine on the incidence rate of the infectious diseases mentioned earlier in Poland.
- Awareness of the impact of the influx of immigrants on the epidemiological situation in Poland.
- Age.

**Figure 3:**
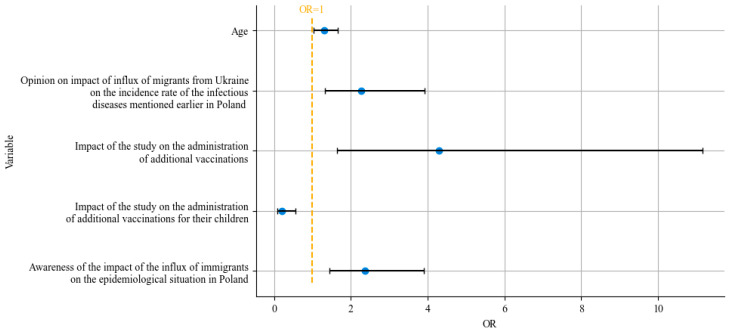
Impact of variables that were identified as significant during statistical analysis on the predictions of the logistic model. Higher values of variables whose odds ratio (OR) exceeded 1 corresponded with a higher chance of answering all of the knowledge questions correctly, according to the model.

The influence of variables mentioned above on the predictions of the model is listed in Table 2. As seen in Figure 3, of the statistically significant variables, only ‘Impact of the study on the administration of additional vaccinations for their children’ had a negative relationship with the dependent variable; all other relationships were positive.

## 4. Discussion

In the study by Ganczak M. et al., none of the ten interviewed Ukrainian women had heard of the *human papillomavirus* (HPV) vaccine. However, after being properly informed, some of them confirmed that they would consider having their daughters vaccinated [14]. When surveying Polish parents in our previous study, we showed that only 9.87% of surveyed chose to give their children additional vaccinations due to migration [19]. Bell S. et al., in a study involving the Polish and Romanian communities living in the United Kingdom, showed that the problem of vaccination uptake is mainly due to problems of communication and access to reliable vaccination information in the respondents’ native languages [20]. In our study, people with greater knowledge of vaccination and the epidemiological situation were more likely to be aware of the potential impact of Ukrainian migration on the epidemiological situation in Poland, compared to those with lower level of knowledge (164/233, 70.39% vs. 147/314, 46.82%, *p* < 0.00001) and were significantly more likely to believe that the influx would increase the incidence of infectious diseases in Poland (200/233, 85.84% vs. 196/314, 62.42%, *p* < 0.00001). Parents who believed that the influx of immigrants will increase the incidence of infectious diseases in Poland were, in general, more knowledgeable in the context of this study (OR 2.28, [CI 1.32–3.92], *p* = 0.003). Having considered the above evidence, it is crucial that healthcare professionals should routinely explain to unvaccinated persons in detail, and above all explain, in an understandable way, the individual and population risks associated with non-vaccination. For this purpose, web portals, brochures, and social media information posted in Ukrainian can be used. The higher the knowledge about vaccination, probably the higher the vaccination coverage in a population. Efendi D. et al. demonstrated that people with higher levels of knowledge are more likely to adhere to vaccination against SARS-CoV-2. Lack of knowledge about vaccination can lead to misperceptions and misunderstandings of vaccination [21]. Similar conclusions were reached by Birmingham et al., who demonstrated that people who lacked knowledge about HPV and the HPV vaccine were more likely to be unvaccinated than those who had a greater level of knowledge about the virus [22]. It was also shown that those with better knowledge, were more likely to believe that COVID-19 could be counteracted by vaccination against SARS-CoV-2 (OR = 1.59, *p* = 0.001) and that everyone should be vaccinated (OR = 4.16, *p* < 0.001) [23]. However, it is important to note that education level, a factor often taken into account in studies, is a different one and that it is not always positively correlated with vaccination knowledge. Nevertheless, Šljivo A. et al. showed that having a bachelor’s degree or higher (OR = 1.61, *p* = 0.006) is associated with a higher level of knowledge on COVID-19 [23]. Kraśnicka J. et al. proved that children of respondents with a higher education are significantly more likely to be vaccinated with recommended vaccines, compared to children of parents with a vocational education (59.5% vs. 36.0%, *p* = 0.016). However, the same study found that parents surveyed with a vocational education were significantly more likely than those with a university education to support the view that vaccination against all diseases was needed (64.0% vs. 18.4%, *p* < 0.00005) [24]. Dąbek J. et al. proved the existence of a positive correlation between the uptake of at least one recommended vaccine and the level of education; those with higher education were significantly more likely, compared to those with primary education, to have received at least one recommended vaccine (53.29% vs. 35.29%, *p* = 0.004) [25]. Vardavas C. et al. also reported that respondents’ educational level and higher socio-economic status were associated with greater knowledge of vaccination [26]. In our study, there were no significant differences in the correlation between the level of education and the number of correct answers regarding knowledge about vaccination and the epidemiological situation (*p* = 0.706). Meanwhile, it was proven that the respondents who had better knowledge about vaccination and the epidemiological situation were more likely to have a positive attitude towards recommended vaccination (213/233, 93.13% vs. 269/314, 85/67%, *p* = 0.040), were more likely to be vaccinated against SARS-CoV-2 (219/233, 93.99% vs. 276/314, 87.9%, *p* = 0.014), and were more likely to vaccinate their children against SARS-CoV-2 (194/233, 83.26% vs. 222/314, 70.70%, *p* < 0.001). One demographic factor that can also influence the level of knowledge of respondents is their age and place of residence. Adella G. et al. showed in their study that people aged ≥49 years were 1.6 times more likely to have good knowledge of the COVID-19 vaccine compared to the 18–34 age group (OR = 1.643) [27]. Raciborski F. et al. showed that confidence in the COVID-19 vaccine and willingness to be vaccinated increased with age, which presumably may be due to their greater knowledge of vaccination [28]. While Vardavas C. et al. found that the more urban the place of residence, the higher the level of knowledge, they did not show the same relationship regarding age [26]. In our study, there was no correlation between place of residence and level of knowledge, while the analysis of the results in the logistic regression indicated that older parents had a higher chance of answering the questions correctly (OR 1.31 [CI: 1.03–1.67], *p* = 0.029). Many studies indicate that parents who intended to be vaccinated against the SARS-CoV-2 virus during the COVID-19 pandemic were more likely to have their children vaccinated as well [29,30,31]. Aldakhil H. et al. have shown that there is a significant correlation between reluctance to vaccinate and low levels of education [32]. However, analysis of the results were yielded by the analysis of the influence of questions ‘Impact of the study on the administration of additional vaccinations’ and ‘Impact of the study on the administration of additional vaccinations for their children’ on the predictions of the model proved surprising. When asked if the influx of immigrants influenced their vaccination decisions, parents who indicated that they plan on or they already have undergone additional vaccinations had a significantly higher chance of answering all the questions correctly (OR 4.29 [1.65–11.17]). Conversely, parents who indicated those answers when asked the same questions in relation to the vaccination of their children had a smaller chance of answering all the questions correctly (OR 0.21 [0.08–0.25]). While the direct reason for this discrepancy is ambiguous, it is possible that it stems from the fact that the children have already been vaccinated with the recommended vaccinations, which is why the parents in this question answered in the negative. However, it should be noted that this result may be the subject of further research. The legal and ethical issues surrounding the vaccination of children are also worth mentioning at this point. The vaccination of a child currently requires the consent of both parents, who may or may not be in favor of its administration. Given that the course of many diseases, such as COVID-19 for example, can be severe in children, and that the willingness to vaccinate may differ among both parents and the child, the issue of vaccination of minors may become a matter of possible legislative discussion in the near future [33].

## 5. Limitations

This study was conducted using an online questionnaire, which did not verify the veracity of the data provided by respondents. The questions regarding additional vaccinations directly verified the willingness to receive additional vaccinations only among those who had not been vaccinated with all available vaccines before participating in the survey.

The performance of the model could be amplified by softening the criteria for being categorized as ‘knowledgeable’. In this approach, people who had six and those who had zero correct answers were both categorized as ‘unknowledgeable’. This threshold of the binary ‘all-or-none’ approach could introduce bias into the results of the study and make it more difficult for the model to provide reliable results.

Another limitation of the study is that the questionnaire was completed during one of the last phases of the health alert linked to COVID-19. On the one hand, feeling fatigued as a result of the prolonged pandemic may have influenced negative responses regarding vaccination, while on the other hand, widely available information may have influenced more informed decisions by parents. Additionally, due to the decrease in cases and hospitalizations, parents may have perceived COVID-19 as less of a threat, which may have also influenced their responses.

However, there are a few strengths worth mentioning, which include the large group of respondents, as well as results that may indicate that there is space for improvement in vaccination rates for a number of infectious diseases.

## 6. Conclusions

Parents with better knowledge of the epidemiological situation in Poland and Ukraine were more likely to take action to reduce the risk of infection with communicable diseases and were aware of the epidemic risk posed by the influx of migrants from Ukraine.

The logistic regression model has provided modest capability in differentiating between parents that answered all of the questions correctly from those who made at least one mistake. Notably, it struggled with identifying parents who answered all of the questions correctly. The regression results confirmed the findings regarding the age of the respondents and their awareness of the impact of Ukrainian migration.

In addition, people who have decided to get additional vaccinations for themselves due to migration, but who have not expressed such a desire for their children, are more likely to be aware of the epidemiological situation in Poland and Ukraine.

## Figures and Tables

**Table 1:** Breakdown of answers in the demographic and opinion sections of the questionnaire depending on the correctness of the answers in the knowledge section.

Characteristic	Total N = 547	Correct Answers to Knowledge Questions N = 233	At Least One Wrong Answer in Knowledge Questions N = 314	*p*-Value
Age in years. median [IQR *]	34 [31–39]	35 [31–39]	34 [30–38]	0.033
Female sex, n (%)	503 (91.96)	214 (91.85)	289 (92.04)	0.935
Place of residence, n (%)				
Rural areas	137 (25.05)	62 (26.61)	75 (23.89)	0.477
City < 50,000	81 (14.81)	37 (15.88)	44 (14.01)	
City 50,000–100,000	67 (12.25)	30 (12.88)	37 (11.78)	
City 100,000–500,000	83 (15.17)	28 (12.02)	55 (17.52)	
City > 500,000	179 (32.72)	76 (32.62)	103 (32.80)	
Education, n (%)				
Primary	1 (0.18)	0	1 (0.32)	
Vocational	5 (0.91)	1 (0.43)	4 (1.27)	0.706
Secondary	55 (10.05)	24 (10.30)	31 (9.87)	
Currently studying	14 (2.56)	5 (2.15)	9 (2.87)	
Higher	472 (82.29)	204 (87.55)	268 (85.35)	
Attitude toward vaccination, n (%)				
Positive	497 (90.86)	217 (93.13)	280 (89.17)	0.277
Negative	7 (1.28)	2 (0.86)	5 (1.59)	
Neutral	43 (7.86)	14 (6.01)o	29 (9.24)	
Opinion on the usage of recommended vaccination, n (%)				
In favor on usage of recommended vaccination, n (%)	482 (88.12)	213 (93.13)	269 (85.67)	0.040
Were vaccinated against SARS-CoV-2, n (%)				
4 doses	99 (18.10)	52 (22.32)	47 (14.97)	0.014
3 doses	266 (48.63)	113 (48.50)	153 (48.73)	
2 doses	115 (21.02)	51 (21.89)	64 (20.38)	
1 dose	15 (2.74)	3 (1.29)	12 (3.82)	
None	52 (9.51)	14 (6.01)	38 (12.10)	
Vaccination of children according to guidelines				
Child vaccinated according to the guidelines, n (%)	537 (98.17)	231 (99.14)	306 (97.45)	0.145
Child vaccinated with recommended vaccines, n (%)				
Yes	448 (81.90)	193 (82.83)	255 (81.21)	0.754
No	91 (16.64)	36 (15.45)	55 (17.52)	
Unknown	8 (1.46)	4 (1.72)	4 (1.27)	
Vaccination of children against SARS-CoV-2				
Child vaccinated against SARS-CoV-2, n (%)	416 (76.05)	194 (83.26)	222 (70.70)	<0.001
Opinion on the impact of the influx of migrants from Ukraine on the incidence rate of the infectious diseases mentioned earlier in Poland, n (%)				
Incidence rate will be higher	396 (72.39)	200 (85.84)	196 (62.42)	<0.001
There will be no differences	151 (27.61)	33 (14.16)	118 (37.58)	
Incidence rate will be lower	0	0	0	
Impact of migration on vaccination decisions, n (%)				
Already vaccinated	26 (4.75)	15 (6.44)	11 (3.50)	0.152
Wanted to get vaccinated	29 (5.30)	15 (6.44)	14 (4.46)	
No impact	492 (89.95)	203 (87.12)	289 (92.04)	
Impact of migration on child vaccination decisions, n (%)				
Already vaccinated	27 (4.94)	12 (5.15)	15 (4.78)	0.363
Wanted to get vaccinated	27 (4.94)	15 (6.44)	12 (3.82)	
No impact	493 (90.13)	206 (88.41)	287 (91.40)	
Awareness of the impact of the influx of immigrants on the epidemiological situation in Poland, n (%)				
Declared awareness	311 (56.86)	164 (70.39)	147 (46.82)	<0.001
Declared knowledge about the situation, but no concern	85 (15.54)	22 (9.44)	63 (20.06)	
Lack of awareness	151 (27.61)	47 (20.17)	104 (33.12	
Impact of the study on the administration of additional vaccinations				
Our study prompted them to think about doing additional vaccinations, n (%)	205 (37.48)	95 (40.77)	110 (35.03)	0.170
Our study prompted them to think about doing additional vaccinations for their children, n (%)	206 (37.66)	83 (35.62)	123 (39.17)	0.397

* IQR—interquartile range.

**Table 2:** Impact of variables that were identified as significant during statistical analysis on the predictions of the logistic regression model. The values shown correspond to importance assigned by the logistic regression model.

Question	OR *	95% CI	*p*-Value
Age [Unit = 1 year]	1.31	1.03–1.67	0.029
Impact of the study on the administration of additional vaccinations for their children	0.21	0.08–0.55	0.002
Impact of the study on the administration of additional vaccinations	4.29	1.65–11.17	0.003
Opinion on the impact of the influx of migrants from Ukraine on the incidence rate of the infectious diseases mentioned earlier in Poland	2.28	1.32–3.92	0.003
Awareness of the impact of the influx of immigrants on the epidemiological situation in Poland	2.38	1.45–3.90	<0.001

* OR—odds ratio, 95% CI—confidence interval.

## Data Availability

The data sets used and/or analyzed during the current study can be made available by the corresponding author upon reasonable request.

## References

[1] Roberts L. (2022). Surge of HIV, tuberculosis and COVID feared amid war in Ukraine. Nature.

[2] Rzymski P., Fulfushynska H., Fal A. (2022). Vaccination of Ukrainian Refugees: Need for Urgent Action. Clin. Infect. Dis..

[3] Cojocaru E., Cojocaru C., Cojocaru E., Oancea C.I. (2022). Health Risks During Ukrainian Humanitarian Crisis. Risk Manag. Healthc. Policy.

[4] Duszczyk M.K.P. (2022). The War in Ukraine and Migration to Poland: Outlook and Challenges. Intereconomics.

[5] Control ECFDPA (2022). Operational public health considerations for the prevention and control of infectious diseases in the context of Russia’s aggression towards Ukraine. Stockholm.

[6] Czarkowski M., Staszewska-Jakubik E., Wielgosz U. (2022). Zachorowania na Wybrane Choroby Zakaźne w Polsce od 1 Stycznia do 31 Grudnia 2021 r. oraz w Porównywalnym Okresie 2020 r.—Biuletyn PZH-NIZP i GIS. http://wwwold.pzh.gov.pl/oldpage/epimeld/2022/INF_22_12B.pdf.

[7] Patel S.S., Moncayo O.E., Conroy K.M., Jordan D., Erickson T.B. (2020). The Landscape of Disinformation on Health Crisis Communication During the COVID-19 Pandemic in Ukraine: Hybrid Warfare Tactics, Fake Media News and Review of Evidence. JCOM J. Sci. Commun..

[8] Cholewik M., Stępień M., Eksmond M., Piotrowska A., Sokołowska M., Bieńkowski C., Pokorska-Śpiewak M. (2023). Measles Complications in Pediatric Patients in Poland. Pediatr. Infect. Dis. J..

[9] Holt E. (2022). Tuberculosis services disrupted by war in Ukraine. Lancet Infect. Dis..

[10] Troiano G., Torchia G., Nardi A. (2022). Vaccine hesitancy among Ukrainian refugees. J. Prev. Med. Hyg..

[11] Giambi C., Del Manso M., Marchetti G., Olsson K., Adel Ali K., Declich S. (2019). Immunisation of migrants in EU/EEA countries: Policies and practices. Vaccine.

[12] Gorman D.R., Bielecki K., Willocks L.J., Pollock K.G. (2019). A qualitative study of vaccination behaviour amongst female Polish migrants in Edinburgh, Scotland. Vaccine.

[13] Bachmaha M. (2016). Vaccination Crisis in Ukraine: Its Origins and Consequences. Krytyka Magazine.

[14] Ganczak M., Bielecki K., Drozd-Dąbrowska M., Topczewska K., Biesiada D., Molas-Biesiada A., Dubiel P., Gorman D. (2021). Vaccination concerns, beliefs and practices among Ukrainian migrants in Poland: A qualitative study. BMC Public Health.

[15] Smith L.E., Amlôt R., Weinman J., Yiend J., Rubin G.J. (2017). A systematic review of factors affecting vaccine uptake in young children. Vaccine.

[16] Dubé E., Laberge C., Guay M., Bramadat P., Roy R., Bettinger J. (2013). Vaccine hesitancy: An overview. Hum. Vaccin. Immunother..

[17] Program Szczepień Ochronnych na 2023 rok 2023. https://www.gov.pl/web/gis/program-szczepien-ochronnych-na-2023-rok.

[18] Social Science Statistics. https://www.socscistatistics.com/.

[19] Cholewik M., Stępień M., Bieńkowski C., Pokorska-Śpiewak M. (2023). Parents’ Attitudes towards Vaccinations Regarding the Ukrainian Migration to Poland in 2022. Vaccines.

[20] Bell S., Edelstein M., Zatoński M., Ramsay M., Mounier-Jack S. (2019). ‘I don’t think anybody explained to me how it works’: Qualitative study exploring vaccination and primary health service access and uptake amongst Polish and Romanian communities in England. BMJ Open.

[21] Efendi D., Rifani S.R., Milanti A., Efendi F., Wong C.L., Rustina Y., Wanda D., Sari D., Fabanjo I.J., De Fretes E.D. (2022). The Role of Knowledge, Attitude, Confidence, and Sociodemographic Factors in COVID-19 Vaccination Adherence among Adolescents in Indonesia: A Nationwide Survey. Vaccines.

[22] Birmingham W.C., Macintosh J.L.B., Vaughn A.A., Graff T.C. (2019). Strength of belief: Religious commitment, knowledge, and HPV vaccination adherence. Psychooncology.

[23] Šljivo A., Abdulkhaliq A., Granov N., Reiter L., Mahendran E., Zeglis I., Abdulkadir Mohammed M., Yousef A., Dadić I., Ivanović K. (2023). COVID-19 vaccination knowledge, attitudes and practices among the general population of Romania during the third wave of COVID-19 pandemic. SAGE Open Med..

[24] Kraśnicka J., Krajewska-Kułak E., Klimaszewska K., Cybulski M., Guzowski A., Kowalewska B., Jankowiak B., Rolka H., Doroszkiewicz H., Kułak W. (2018). Mandatory and recommended vaccinations in Poland in the views of parents. Hum. Vaccin. Immunother..

[25] Dąbek J., Sierka O., Gąsior Z. (2022). Protective vaccinations in the control and prevention of infectious diseases—Knowledge of adult Poles in this field. Preliminary results. BMC Public Health.

[26] Vardavas C., Nikitara K., Odani S., Symvoulakis E. (2022). The predictors and association between knowledge of vaccines and vaccination among adults and children in 28 European Countries, 2019. Popul. Med..

[27] Adella G.A., Abebe K., Atnafu N., Azeze G.A., Alene T., Molla S., Ambaw G., Amera T., Yosef A., Eshetu K. (2022). Knowledge, attitude, and intention to accept COVID-19 vaccine among patients with chronic diseases in southern Ethiopia: Multi-center study. Front. Public Health.

[28] Reczulska A., Tomaszewska A., Raciborski F. (2022). Level of Acceptance of Mandatory Vaccination and Legal Sanctions for Refusing Mandatory Vaccination of Children. Vaccines.

[29] Temsah M.H., Alhuzaimi A.N., Aljamaan F., Bahkali F., Al-Eyadhy A., Alrabiaah A., Alhaboob A., Bashiri F.A., Alshaer A., Temsah O. (2021). Parental Attitudes and Hesitancy About COVID-19 vs. Routine Childhood Vaccinations: A National Survey. Front. Public Health.

[30] Wang Q., Xiu S., Zhao S., Wang J., Han Y., Dong S., Huang J., Cui T., Yang L., Shi N. (2021). Vaccine Hesitancy: COVID-19 and Influenza Vaccine Willingness among Parents in Wuxi, China—A Cross-Sectional Study. Vaccines.

[31] Teasdale C.A., Borrell L.N., Shen Y., Kimball S., Rinke M.L., Fleary S.A., Nash D. (2021). Parental plans to vaccinate children for COVID-19 in New York city. Vaccine.

[32] Aldakhil H., Albedah N., Alturaiki N., Alajlan R., Abusalih H. (2021). Vaccine hesitancy towards childhood immunizations as a predictor of mothers’ intention to vaccinate their children against COVID-19 in Saudi Arabia. J. Infect. Public. Health.

[33] Marrone M., Luca B.P.D., Stellacci A., Buongiorno L., Caricato P., Cazzato G., Ferorelli D., Solarino B., Stefanizzi P., Tafuri S. (2022). COVID-19 Vaccination in Italian Children: The Limits of Parental Rights. Children.

